# New life sciences innovation and distributive justice: rawlsian goods versus senian capabilities

**DOI:** 10.1186/2195-7819-9-5

**Published:** 2013-05-28

**Authors:** Theo Papaioannou

**Affiliations:** ESRC Innogen Centre and Development Policy and Practice, The Open University, Milton Keynes, UK

## Abstract

The successful decoding of human genome and subsequent advances in new life sciences innovation create technological presuppositions of a new possibility of justice i.e. the just distribution of both social (income, wealth, etc.) and natural (rationality, intelligence, etc.) goods. Although Rawlsians attempt to expand their theory to include this new possibility, they fail to provide plausible metrics of social justice in the genomics and post-genomics era. By contrast, Senians seem to succeed to do so through their index of basic capabilities. This paper explores what might be regarded as a Senian perspective of distributive justice in new life sciences innovation. The argument is that, by comparing freedoms (different functionings) instead of primary goods, the capability theory allows not only for the identification of injustices linked to natural lottery but also for their elimination through the use of new genomic technologies, including gene-based diagnostics, gene therapy, somatic cell engineering (SCE) and germ-line engineering (GLE). These innovative technologies seem to have the potential to reduce variability in natural goods and therefore enable individuals to convert social goods into well-being or welfare.

## Introduction

Recent advances in new life sciences innovation, including new diagnostic tools and genetic interventions (Propp & Moors 2009), challenge the traditional idea of justice according to which morally arbitrary natural inequalities are given facts that remain outside the domain of justice. This idea of justice can be traced to John Rawls’s *A Theory of Justice*(1971) that is one of the most influential works in moral and political philosophy. Rawls advanced two principles of justice, the so called ‘equal liberty’ and the ‘difference principle’, in order to assess the basic structure of society in terms of an index of social primary goods such as rights, liberties, opportunities, income and wealth, and the social bases of self-respect (Rawls 1999). However, new life sciences innovation creates technological preconditions for a new possibility of justice; namely the just distribution of both social primary goods (income, wealth, etc.) and natural primary goods (rationality, intelligence, etc.). Although contemporary followers of Rawls (thereafter Rawlsians) such as Farrelly (2004) and Buchanan et al. (2000) attempt to expand their theory to include this new possibility, they fail to provide plausible metrics of social justice in the genomics and post-genomics era. By contrast, Amartya Sen (2009) and his followers (thereafter Senians) such as Fox (2007) and Cooke (2003) seem to succeed to do so through their index of basic capabilities. Sen introduced his capability approach as an alternative to Rawls’s theory of justice. His focus is no longer on primary goods but on what people are actually able to do and be, having freedom to achieve different functionings. This shift of focus makes the capability approach more open to new life sciences innovation.

This paper critically explores what might be regarded as a Senian perspective of distributive justice in new life sciences innovation. The argument is that, by comparing freedoms (different functionings) instead of primary goods, the capability approach allows not only for the identification of injustices linked to natural lottery but also for their elimination through the use of new genomic technologies, including gene-based diagnostics, gene therapy, somatic cell engineering (SCE) and germ-line engineering (GLE). Although these innovative new technologies are still under development, they seem to have the potential to reduce variability in natural goods and therefore enable individuals to convert social goods into well-being or welfare.

This paper is structured as follows: firstly we discuss the failure of Rawlsians to provide plausible metrics of social justice in the genomics and post-genomics era; secondly, we examine the advantages and disadvantages of a Senian perspective of distributive justice in new life sciences innovation; finally we conclude that Sen’s theory, properly interpreted, is likely to succeed in providing plausible metrics of justice in the new technological era through an index of basic capabilities.

### The problem of rawlsians in the genomics and post-genomics Era

Since the completion of the Human Genome Project (HGP), it has been hard to keep up with the pace of innovative developments in new life sciences (i.e. genomics and post-genomics as opposed to genetics) let alone their applications to key sectors of society and economy such as human health. In terms of specific technologies, genomics and post-genomics offer the prospect of development of new gene-based diagnostics (e.g. pre-implantation genetic diagnosis or PGD) and identification of underlying genetic components of diseases. These innovations are no longer of futuristic kind; they have already become actualised and well established (Denier 2010). Also the introduction of personalised medicine promises treatments designed for individual patients (i.e. pharmacogenetics) while the development of gene- and cell-based therapies, including gene therapy and the use of stem cells, create expectations not only for preventing and/or curing diseases but also for enhancing human beings’ traits and capacities (Gottweis 2002). Genetic enhancements are envisaged through new life sciences innovation, especially by manipulating DNA and rewriting genetic codes through somatic cell engineering (SCE) and germ-line engineering (GLE)^a^. These innovative technologies promise alteration of the genes in all cells in the human body. Finally, the development of synthetic biology promises to design and engineer biologically based parts of novel systems as well as redesigning existing natural biological systems.

As has been already argued elsewhere (Papaioannou 2009), the 21^st^ century life sciences innovation offers the technological basis of changing prior assumptions about human nature and thereby introducing a new possibility of justice that goes beyond the distribution of social goods (e.g. income, wealth, etc.)^b^. Indeed, the ability to manipulate DNA and rewrite genetic codes, as well as the possibility of applying engineering principles to design new biological systems (i.e. synthetic biology), have increased the prospects of intervention in the natural lottery and therefore have changed our perceptions of natural goods (e.g. health, vigour, intelligence, imagination, etc.) from matters of chance to matters of choice (Buchanan 2000). To put it another way, some (morally significant) natural goods, previously perceived as matters of chance, may soon be distributable and therefore become matters of choice. As Buchanan et al. (2000) argue

If precise and safe control over the distribution of natural assets becomes feasible, then those who believe that justice is concerned with effects of natural assets on individuals’ life prospects will no longer be able to assume that justice requires only that we compensate for bad luck in the natural lottery than attacking natural inequalities directly.

Given this new possibility thanks to new life sciences innovation, we must indeed ‘…begin to take seriously the idea that charges of *fairness* and *unfairness* can apply to the distribution of genetic endowments (italics added) (Farrelly 2004).

Although some scholars (Pence 1998;Cooke 2003;Fox 2007) argue that Rawls allows for the equalisation of natural goods through genetic interventions, some others (Farrelly 2004) claim that his theory needs to be extended in order to address the new possibility of justice in the genomics and post-genomics era. In any case, Rawlsians believe that they have grounds to be concerned with variations in natural goods when these are not to the greatest benefit of the least advantaged. Like all egalitarians, Rawlsians argue that peoples’ advantage should not be determined by pure bad (brute or optional) luck (Barclay 2009). Those who are born with poor natural endowments may be further disadvantaged if they cannot access life science innovations to modify or boost them. Consider the argument of Colin Farrelly. He urges us to ‘…start thinking about what demands of justice will be in the post-genetic revolutionary society’ (Farrelly 2004). Farrelly is a Rawlsian thinker in two respects: first, he acknowledges the importance of primary goods for the theory of justice as fairness; second, he follows Rawls in dividing such goods into *social primary goods* (SPG) such as rights and liberties, powers and opportunities, income and wealth, and *natural primary goods* (NPG) such as health, vigour, intelligence and imagination.

Both SPG and NPG are things that people are presumed to want (Rawls 1982). Although there is a clear relationship between them (e.g. there is clear correlation between good health and high income and wealth in non-welfare societies), Farrelly (2002) correctly observes that ‘Rawls general conception of justice stipulates that only the SPG are to be equally distributed, unless unequal distribution of any or all of these values is to everyone’s advantage. But what about the NPG? Why does Rawls not include the distribution of them in the principles of justice?’ Farrelly’s answer is this: in Rawls’s theory, the possession of NPG is influenced by the basic structure. Nevertheless, these goods are not under the direct control of the basic structure. Farrelly argues that ‘This point is of the utmost importance when considering how advances in genetic research will revolutionise debates concerning social justice. For these technologies will make it possible for the distribution of NPG to be directly (though not totally) under our control’ (Farrelly 2004). Take for example PGD and gene therapy. The fact that gene screening is now widely available and gene therapy is improving increases control over the genetic constitution of our children. On this basis Farrelly (ibid) proposes a revised theory of justice as fairness that incorporates Rawlsian principles to govern the just distribution of NPG. His starting point is the following: ‘It is no longer viable to invoke the standards of social justice from a world where it was only the SPG that were directly under the control of the basic structure’.

Farrelly is convinced that the future society will be one where the distribution of NPG will be, to a large extent, under the control of the basic structure. But of course, as (Denier 2010) stresses ‘…not everything we can control is a matter of justice or injustice. As such, control is a necessary but not a sufficient condition for justice’. It is necessary to justify that the NPG controlled by the basic structure are so important for people that they become concerns for justice. But who decides whether health, vigour, intelligence and imagination are NPG so important that they become concerns of justice? The presumption that everyone wants them is philosophical and does not provide full justification. To achieve full justification would also require some empirical evidence that, for example, lack of NPG such as intelligence and imagination makes everyone disadvantaged. On this issue Farrelly appears to be silent. Instead he raises the issue of principles which should govern the just distribution of NPG.

As an answer, (Farrelly 2004) (ibid: 81) proposes a ‘genetic difference principle’ (GDP).

This principle states: inequalities in the distribution of genes important to the NPG are to be arranged so that they are to be the greatest benefit of the least advantaged.

Farrelly explicitly recognises that total control of the distribution of NPG would be impossible. What would be possible is advances in new life sciences innovation would enable the ‘fair’ distribution of genes (i.e. genetic justice) and this would influence NPG. It might be argued that there are several problems with the application of Farrelly’s GDP. First of all, the maintenance of distinction between NPG and SPG overlooks the sociological fact that human beings are anyway reproduced through some sort of ‘selective breeding’. For example, social, political and economic factors influence who marries and reproduces with whom, and thereby determines the range of genetic endowments. Also social choices determine the size, shape and gender balance of populations and therefore also NPG. The ability to offer genetic interventions by means of new life sciences innovation is yet another place of social influence into the genetic endowments of human beings^c^.

Secondly, even if we assume that Farrelly’s GDP is plausible and applicable, it is not clear which individuals qualify as members of the worst-off group of society (Papaioannou 2009). For instance, Lindsay (2005) insists that severely cognitively impaired individuals qualify as members of the least advantaged group and for this reason they should be given priority over other genetically disadvantaged groups. But it is not clear why this should be the case. Why should some genetically disadvantaged group be given priority over other genetically disadvantaged groups? Nor is it clear why victims of genetic diseases should be given priority over victims of socio-economic circumstances (e.g. those born in poverty). Farrelly’s reply to prioritarians such as Lindsay is that the interests of different disadvantaged groups need to be balanced one against another. The GDP is not strict and therefore does not trump those principles that govern the distribution of SPG.

Farrelly allows for a more ‘lax’ interpretation of GDP.

This interpretation stipulates that genetic inequalities should be arranged so that they are to the greatest *reasonable* benefit of the least advantaged (Farrelly 2004).

In fact, the lax GDP compromises genetic justice against other legitimate claims of justice. In addition, society should decisively deal with the issue of ‘which genetically disadvantaged group should be given priority over other genetically disadvantaged groups’. Farrelly’s argument is plausible but fails to address the epistemological problem that is at the heart of prioritarian investigations: how can we know who is the least advantaged in terms of NPG? Farrelly (2008) offers the strategy to define the ‘least advantaged’ as ‘…those individuals whose genetic constitutions place them below half of the median for expected life time acquisition of natural primary goods’. This strategy does not focus on individual genetic constitutions but rather on their consequences for the acquisition of natural primary goods. It might be argued that there are two problems with Farrelly’s strategy: first of all, it is not clear how the expected life time acquisition of natural primary goods will be determined (expectations might vary across different societies); secondly, Farrelly’s strategy focuses on the consequences of genetic constitutions and not on genetic constitutions as such (this means it ignores the possibility of non-genetic determinism i.e. acquisition or non-acquisition of natural primary goods mainly as a result of social and economic conditions).

Thirdly, the lax GDP considers ‘genes’ and genetic endowments to be (genetic) resources. This is mistaken for two reasons. Firstly, equalisation of genetic resources might leave people who live in poor economic and social conditions (e.g. poverty) worse off than others because of negative gene-environment interactions. Secondly, as Cooke points out, ‘If genetic endowments are interpreted as resources, one may be inclined to redistribute as much as possible and be as ‘charitable’ as possible, going well beyond disease prevention, and into enhancement, where height, good looks, physical strength and intelligence, would be considered generous endowments of resources for one’s future descendants (Cooke 2003).’ The problem is that one’s genes and genetic endowments cannot easily change. If they change by means of new life science innovations such as GLE they may affect future generations. But, of course, one generation cannot know which genetic endowments or traits would be best for future generation simply because one cannot predict what the conditions would be in the future. According to Cooke ‘…the further moral problem is that in enhancing these ‘goods’, parents make choices for future generations by casting their own values into their world’ (Cooke 2003).

Fourthly, the lax GDP considers ‘genes’ to be the only plausible currency of genetic justice without taking account of the biological and conceptual complexities involved in the distribution of genes. As Fox stresses, complex traits, including health, vigor, intelligence and imagination, ‘…are embedded and developed through robust interplay of heredity and environment and their biological input is a complex function of multiplicity relating genes, which is highly unlikely to be directly manipulable through biotechnology intervention at any point in the near future. Beyond the causal complexities of multigenecity and gene-environment interaction, there remain conceptual complexities as to both the socio-economic desirability of a particular trait within a given system of social arrangements, and also the moral desirability of a trait by reference to the plurality of worldviews with that system’ (Fox 2007). The complexity of genes (and especially their interaction with the external environment), disqualify them from being the currency of new distributive justice. This implies that the lax GDP is impossible to be applied, equalising genes that are important to NPG.

### A senian perspective of distributive justice in New life sciences innovation

If it is true that Rawlsians such as Farrelly fail to provide plausible metrics of justice (i.e. genes important to NPG distributed according to the lax GDP) in the genomics and post-genomics era, is it also true that Senians such as Cooke (2003) and Fox (2007) succeed to provide better metrics through the capabilities approach? If the answer is yes then what might be regarded as a Senian perspective of distributive justice in the era of new life science innovation?

In the *Tanner Lectures on Human Values*, Sen (1980) asserts that what matters in the discussion of social justice is not the question ‘why equality’ but the question ‘equality of what?’ Answering this latter question provides the currency of Senian justice which is capability. The capability approach shifts focus from the basic structure of society to the basic freedoms of people. Sen is critical of Rawlsian principles because they become central issues in judging resource-based distributional equity. In this sense, he suggests there is an element of ‘fetishism’ in those principles (Sen 1980). In Sen’s view, resources are merely the means to freedom for people to choose different kinds of life. As Sen (1980) explains:

Justice cannot be indifferent to the lives that people can actually live. The importance of human lives, experiences and realisations cannot be supplanted by information about institutions that exist and the rules that operate … We have reason to be interested … in the freedoms that we actually have to choose between different kinds of lives.

This is what the notion of capability refers to: each individual’s actual freedom to achieve the functioning he/she values (leading an autonomous and meaningful life). Sen arrives at the notion of capability partly through his critical assessment of Rawlsian primary goods. Sen’s dissatisfaction with Rawlsian primary goods is founded upon the notion that some individuals are differently capable of converting them into well-being or welfare because of variations in a range of factors, including biology, physical environment and social conditions. For example, disabled people cannot achieve the same level of well-being as non-disabled people with the same amount of primary goods. Primary goods as such do not matter; they cannot (and should not) be thought as the currency of egalitarian justice (Cohen 1989). What matters is what individuals are able to do with them. For Sen the ultimate concern is not primary goods as such but capabilities which are the result of relations between persons and goods and physical environment, and social conditions. Capabilities imply the importance of the freedom dimension of human life.

The distributive objective of the capability approach is to guarantee all individuals access to the basic abilities required for the achievement of essential functionings so that no one falls below the relevant baseline into what Sen calls a state of ‘capability deprivation’. Above that baseline it seems that individuals bear responsibility for their choice of non-essential functionings. Although Sen prioritises freedom over responsibility, the latter seems to be important for understanding one crucial aspect of the capability approach: individuals who have the capability to achieve essential functionings but choose not to do so or individuals who choose certain non-essential functionings cannot justify and sustain complains about essential functionings achievement-failure. Such individuals do not lack the capabilities and so there is no reason to complain or transfer resources from those who have chosen essential functionings to those who have chosen non-essential functionings. Even though Sen never takes a clear position on this issue, he defends substantive freedom of choice that can never be separated from responsibility. As he says

Freedom to choose gives us the opportunity to decide what we should do, but with that opportunity comes the responsibility for what we do – to the extent that they are chosen actions. Since the a capability is the power to do something, the accountability that emanates from ability – that power – is a part of the capability perspective … (Sen (1980)

Certainly, to know how many of an individual’s functionings were a result of his/her choice and how many a result of unchosen circumstances, we would have to know details about that person’s situation. Collecting data about a person raises a number of ethical issues, including what Wolff (1998) regards as ‘shameful revelation’ i.e. ‘…one may find certain facts about oneself shameful, and not wish to reveal them’ Sen (1980). Shameful revelation can lead to lower respect-standing and therefore undermine egalitarian ethos. The capability approach does not demand investigation into one’s essential functionings achievement-failure. That is to say, one is not required to admit he/she is a failure because he/she finds it difficult to achieve essential functionings, when there is no difficulty for other, equally capable, individuals.

In Sen’s theory, the notion of responsibility includes the element of voluntariness. Individuals are responsible for the non-essential functionings they have voluntarily chosen (from n-tuples) as capable actors. In fact, Sen assumes responsibility in every individual choice. For this reason he insists that ‘In dealing with responsible adults, it is more appropriate to see the claims of individuals on the society (or the demands of equity or justice) in terms of *freedom to achieve* rather than *actual achievements*’ (Sen 1995). Although freedom as such is not an absolute value in Sen’s theory, it is a value of primary importance or fundamental value. Indeed freedom is the bottom of everything. According to Fleurbay (2006) ‘Typically, even when freedom is deemed important one would still look at achievements as well’. Achievements constitute consequences of the capability approach. Therefore, they cannot be totally ignored by Sen’s evaluative system.

The components of responsibility and freedom within the capability approach make it attractive to those who defend justice in new life sciences innovation. Thus, according to (Cooke 2003) ‘Amartya Sen’s capability theory can be used as a framework to ensure freedom and equality in the use of GLE technology’. The latter is a new life sciences innovation that might be used for genetic interventions (i.e. interventions in human genes) in order to provide the biological basis of certain basic human capabilities. In this sense the focus here is neither on genes nor on NPGs. None of these has an intrinsic value. Instead, the focus here is on the freedom of people to choose among combinations of functionings. Genes and NPGs can help one achieve more freedom. Therefore, they might be only viewed as means to freedom (Sen 1980). But as Sen stresses, means cannot be valued independently of ends (Sen 1980). Since genes and NPGs cannot be valued for their own sake, their dependence on the valuation of capabilities has to be acknowledged. In the case of GLE technology, the valuation of interventions in human genes that influence NPGs depends on the valuation of actual functionings. That is the extent of freedom that can be achieved by means of NPGs (influenced by GLE interventions in human genes).

Certainly, Cooke (ibid) seems to overlook one important issue: Sen’s theory is not necessarily about intergenerational justice (although it does not exclude intergenerational justice) while GLE is about irreversible changes into genetic material and this can affect both future states of well-being and future generations. What if those changes compromise current persons’ ability to secure future states of well-being? What if those changes prove to be undesirable to future persons? The answer seems to be that even if GLE based changes prove to be undesirable for some current and future persons, these persons can still make a responsible choice not to convert them into actual functionings (even if they are capable of doing so). In other words, future generations can responsibly choose to achieve functionings different from those based on undesirable GLE changes. As Wolff (2009) acknowledges it is indeed ‘…possible to have a capability to achieve a functioning, yet choose not to do so’. This is because there is a distinction between functionings and capabilities. Functionings focus on what persons actually choose to achieve. By contrast, capabilities focus on the alternatives persons have (Anand et al. 2005). To be capable of certain functionings does not mean that persons necessarily choose to achieve them (persons might choose from a set of alternatives). Although it is true that we cannot know enough to secure the well-being of future generations, this is not a problem for the capability approach to justice.

Ensuring capabilities through GLE and other new life science technologies is a justice enhancing as it gives people the choice to express their genes, converting them into functionings. This possibility of choice of future generations also makes genetic interventions defensible in the present. The justification being that no matter how desirable or undesirable such interventions will be for future generations, it is the future generations who will make the choice about which to convert into actual functionings.

Basic human capabilities have both biological and social determinants. As (Cooke 2003) stresses ‘Social and environmental influences, personal choices, and hard work should not be underestimated. These factors have always been great competitors to generate predispositions in enabling people to achieve their goals and overcome genetic inequalities’. Indeed, as Wilkinson and Pickett (2009) and Brock (2000) also confirm, where one stands in the socio-economic hierarchy and the degree of socio-economic inequality affect health.

The biological and social determinants of human capabilities imply that a Senian perspective of justice requires provision of a decent minimum of both genetic goods and social goods so that people are able to choose among combinations of functionings. Therefore, it is neither the genetic goods nor the social goods that directly contribute to well-being. Rather, it is the capabilities which constitute and contribute to well-being. For this reason the role of society and government is to provide different means (including life sciences innovation and policies of social and economic redistributon) in order to bring people up to at least a minimum threshold of capabilities. Some people might need serious genetic interventions (by means of new life sciences) in order to arrive at the minimum threshold while some other people might need serious social interventions in order to arrive at the same threshold or even both (genetic and social interventions). The Senian perspective of distributive justice also allows for the gene-environment interaction to be taken seriously on board in order to bring people up to at least a minimum threshold of capabilities. The gene-environment interaction has impact on whether people are capable of achieving certain functionings. For instance, common diseases such as heart disease and cancer depend on this complex interaction and prevent people from being able to achieve good health.

Despite the fact that the Senian perspective deflects attention from natural (primary) and social (primary) goods to what those goods and their interaction do for people, it never specifies what sort of capabilities can be considered as basic capabilities in the genomics and post-genomics era. Sen leaves the notion of basic capabilities open to interpretation. According to him ‘The capability approach is a general approach, focusing on information on individual advantages judged in terms of opportunity rather than specific ‘design’ for how society should be organised’ Sen (1980). This generality and openness forced Senians such as Nussbaum (2000) to provide the following interpersonal and comparable index of basic capabilities:

1. Life. Being able to live to the end of a human life of normal length…
2. Bodily Health. Being able to have good health…
3. **Bodily Integrity.** Being able to move freely from place to place…
4. **Senses, Imagination, and Thought.** Being able to use the senses, to imagine, think and reason…
5. **Emotions.** Being able to have attachments to things and people…
6. **Practical Reason.** Being able to form a conception of the good and to engage in critical reflection…
7. **Affiliation.A.** Being able to live with and toward others, to recognise and show concern for other human beings…**B.** Having the social bases of self-respect and non-humiliation; being able to be treated as dignified being…
8. **Other Species.** Being able to live with concern for and in relation to animals, plants and world of nature..
9. **Play.** Being able to laugh, to play, to enjoy recreational activities…
10. **Control over One’s Environment.A. Political.** Being able to participate effectively in political choices that govern one’s life…**B. Material.** Being able to hold property (both land and movable goods)…

Nussbaum recognises that some items on her philosophically constructed index of capabilities are or include what Rawls called natural primary goods but stresses that the determinants of those goods are natural or luck-governed. In fact, as pointed out elsewhere (Papaioannou 2009), she is not open to (or she ignores) life sciences innovation and therefore she argues that ‘In these areas, what government can aim to deliver is the social basis of these capabilities…’. Nussbaum (2000) takes the example of women’s emotional health. She insists that ‘…government cannot make all women emotionally healthy; but it can do quite a lot to influence emotional health through suitable policies in areas such as family law, rape law and public safety. Something similar will be true for all the natural goods’ Nussbaum (2000).

Nussbaum’s view that government can only be indirectly involved in the distribution of natural or luck governed goods does not seems to go far in the genomics and post-genomics era. What if government came up with suitable policies of equal access to life sciences innovations (including GLE) that could improve the biological bases of natural goods? To put it another way, what if society as a whole could guarantee both the biological and the social bases of basic capabilities?

Most of the basic capabilities in Nussbaum’s index reveal a strong genetic component. As (Fox 2007) observes ‘…they are closely correlated with physical, mental and behavioural goods among the natural primary goods. Physical goods such as absence of disability and resistance against disease, for example, form important genetic basis for capabilities, such as being able to have good health, move about freely, and live out a worthwhile life of normal length’. For Fox a Senian perspective of distributive justice in the genomics and post-genomics era implies a decent genetic minimum that consists of a biological constitution that provides whatever internal components necessary for people to function at adequate level in each of the basic capabilities. Whenever there is a mismatch between biology and basic capabilities, there is a requirement of justice to alter biology (or gene-environmental interactions) so that people can be brought up to adequate level of functioning.

This perspective seems to be more about treatment than enhancement. Treatment and enhancement are difficult to distinguish (Levitt & O’Neill 2010), but a line might be drawn between them for the sake of capability theory. According to Mehlman et al. (2011) enhancement is ‘An intervention that employs medical and biological technology to improve performance, appearance or capability besides what is necessary to achieve, sustain, or restore health’. By contrast, an intervention that aims to treat or mitigate the effects of a disease would qualify as treatment. Certainly, as Green (2005) stresses, between treatment and enhancement lies a third category ‘prevention’. ‘Unlike … treatment… prevention is an intervention that alters an individual’s condition beyond what is normal for the species, but it does so in order to reduce person’s risk of suffering disease’ (Fox 2007). Although both treatment are prevention are based on a controversial approach to disease as a deviation from species-typical functions (Resnik 2006), the list of basic capabilities seems to correspond to these functions. For example, the capability of life corresponds to the human species function or ability to live a life as this has become typical or normal through natural selection (Daniels 1985). From this it follows that healthy people are capable of life. Therefore to perform genetic interventions (by means of life science innovations) to enhance otherwise healthy people is not justified by the outlined Senian perspective of justice. What is justified is genetic intervention to either prevent or treat diseases that result in a state of basic capability deprivation. Although some (Lenox 1995) might argue that such intervention is still a form of enhancement (as indeed all medicine is), the Senian perspective can allow it only if it can bring people above a decent minimum of basic capability deprivation. In this sense, it might be argued that a decent minimum of basic capability deprivation constitutes an equilibrium point. Any genetic intervention beyond that point (aiming at unlimited capability development) can be considered as enhancement and therefore can be rejected on the grounds that it increases inequality. By contrast, any genetic intervention up to that point (aiming at basic capability development) can be considered as treatment and therefore can be promoted on the grounds that it reduces inequality.

According to (Fox 2007) the first principle of the Senian perspective is the following:

1. Natural primary goods are to be distributed so as to (contribute the heredity bases of crucial human functioning that) bring individual off-spring up above a decent minimum of internal basic capability deprivation.

Fox stresses that this first principle is *sufficientarian*. However, it says nothing about inequality of capabilities of those individuals above the decent genetic minimum. In order to go beyond sufficientarianism, he introduces a second principle:

2) Natural primary goods are to be distributed (so as to contribute the heredity base of crucial human functioning that is) to the greatest benefit of the least advantaged.

This second principle is *prioritarian* and supposedly deals with the problem of inequality of capabilities of individuals above the decent genetic minimum. As long as such inequality does not threaten sufficiency, it can be justified within the Senian perspective. In fact, similarly to Farrelly’s GDP, Fox’s prioritarianism ignores physical complexities such as gene-environment interaction.

Although Fox’s hybrid distributive theory correctly identifies the limitations of the basic capability approach, it fails to provide a coherent alternative. There are two main reasons for this: first, it is unclear which primary goods ought to be distributed to the least advantaged after the point at which everyone has been brought above a decent genetic minimum; second, Fox tries to bring together the Rawlsian and the Senian perspectives without acknowledging that each of these perspectives has a its own unique currency of justice.

As has been already said, the capability approach is informational. One might use this approach to evaluate inequalities that have both genetic and social causes but not sign up to specific public policies aimed at equating genetic goods through the use of life sciences innovation. According to Sen (1980)‘…in judging the aggregate progress of society, the capability approach would certainly draw attention to the huge significance of the expansion of human capabilities of all members of the society, but it does not lay down any blueprint for how to deal with conflicts between, say, aggregative and distributional considerations …’. Sen is clear that the informational nature of the capability approach is crucial for making the right decisions about public policy interventions. As he says ‘…the choice of an informational focus – a concentration on capabilities – can be quite momentous in drawing attention to the decisions that would have to be made and the policy analysis that must take account of the right kind of information’ Sen (1980). From this it follows that specific (scientific) information about the relationship between genetic inequality and environment and basic capability deprivation can draw attention to the need for specific decisions and policies for integrated interventions e.g. social interventions through re-distribution of income and genetic interventions through gene therapy or GLE. Such interventions might reduce inequality not so much in SPGs and NPGs, but in ‘…relations between superior and inferior persons’ (Anderson 1999). Properly understood, egalitarianism opposes hierarchical social relations. In her critique of Rawlsians and other luck egalitarians, Anderson clearly stresses that the point about egalitarianism is to ‘…seek a social order in which persons stand in relations of equality. They seek to live together in a democratic community as opposed to hierarchical one’ Sen (1980). By preventing people from arriving at a state of basic capability deprivation through both social and genetic interventions, Senians are likely to succeed eliminating oppressive social and political relations. Redistribution of income, gene therapy or GLE might be crucial for enabling some people to function as equal citizens.

## Conclusion

In this paper, I have tried to explore a Senian perspective of justice in new life sciences innovation. I have argued that, given the failure of Rawlsians to provide plausible metrics of justice in the genomics and post-genomics era, Senians, by comparing freedoms instead of primary goods, allow for the elimination of natural lottery injustices through life science innovation, including GLE. Otherwise these injustices might result in a state of basic capability deprivation. The outlined Senian perspective needs to be further elaborated in order for us to come up with capability based metrics of justice in the genomics and post-genomics era.

## Endnotes

^a^Somatic cells are mainly skin or muscle cells, they contain 23 chromosomal pairs and do not transmit genetic information to future generations. By contrast, germ-line cells are the egg and sperm cells, they contain 23 chromosomes and transmit genetic information to offspring as well as to future generations (see F. Allhoff. Germ-line Genetic Enhancement and Rawlsian Primary Goods. *Kennedy Institute of Ethics Journal* 2005; 15(1): 39–56). Germ-line engineering of the human genome might become technically feasible in less than a decade since some specific techniques already exist and have been demonstrated in animals (see M. Lappé. 2006. Ethical Issues in Manipulating the Human Germ Line. In *Bioethics: An Anthropology*. H. Kuhse and P. Singer eds. Oxford. Blackwell.

^b^This, in fact, sketches a move from ‘fate’ to ‘responsibility’ i.e. responsibility for just distribution of natural goods.

^c^This critical point was made by one of this journal’s referees. I would like to thank him/her for his/her contribution.
